# Trait Acceptance Buffers Aggressive Tendency by the Regulation of Anger during Social Exclusion

**DOI:** 10.3390/ijerph192214666

**Published:** 2022-11-08

**Authors:** Conglian He, Jixuan Mao, Qian Yang, Jiajin Yuan, Jiemin Yang

**Affiliations:** 1 The Affect Cognition and Regulation Laboratory (ACRLab), Institute of Brain and Psychological Sciences, Sichuan Normal University, Chengdu 610066, China; 2 Xi’an Jingkai No.1 School, Xi’an 710000, China

**Keywords:** trait acceptance, aggressive tendency, emotions, social exclusion

## Abstract

Social exclusion has led to increased negative emotions and aggressive behaviors, two outcomes that are correlated with each other. Thus, the down-regulation of negative emotions appears to play a crucial role in reducing the tendency for aggressive behavior. However, this assumption has not yet been tested. To this end, a total of 397 undergraduates reported their aggressive tendencies, state emotions and trait acceptance by completing corresponding questionnaires, and a recall paradigm was used to induce experiences of social exclusion. The results showed that in the context of social exclusion, (1) trait acceptance was negatively correlated with negative emotions and aggressive tendency but was positively correlated with positive emotions; (2) negative emotions, rather than positive emotions, were positively correlated with aggressive tendency; (3) increased trait acceptance buffered the experience of anger, which is, in turn, related to reduced aggressive tendency; (4) trait acceptance also downregulated the feeling of sadness, which is, however, related to increased aggression; (5) the mediator of sadness was smaller in effect size than that of anger. Taken together, these results suggest that negative emotions are associated with aggression in the context of social exclusion, and the habitual use of an acceptance strategy was conductive to decreasing aggressive tendencies by decreasing anger.

## 1. Introduction

Given its strong relationship to psychological well-being, it is important for humans to develop and sustain stable social connections with others. Alternatively, the destruction of social ties would lead to social pain that is comparable to physical pain [1], with social exclusion being a classic example [2]. Social exclusion is the phenomenon in which people are rejected or repelled by another person or group [3], which interferes with their fundamental need for interaction and belonging [4]. Moreover, a large number of studies revealed that social exclusion increases aggressive behaviors [5] in addition to unpleasant emotions, such as sadness, anger and hurt feelings, etc. [6]. For example, it has been shown that social exclusion can predict aggression both in adolescents [7] and college students [8]. Additionally, laboratory studies also supported the notion that people who had been socially excluded exhibited higher levels of aggression toward the individual who initiated the ostracism and innocent bystanders [5]. To promote people’s psychosocial development, it is crucial to effectively reduce negative emotions and aggressive behaviors brought on by social exclusion.

Previous studies from various perspectives, including the use of emotion regulation strategies, have explored ways to reduce negative emotions induced by social exclusion. For example, the effectiveness of reappraisal strategies in down-regulating negative emotions and social pain has been demonstrated in previous studies [9,10]. However, inflexible cognitive reappraisal may cause individuals to deny the significant features of the situation [11], and such denial was found to be related to individual’s hostile cognitive bias and more aggressive responses after being rejected [12]. It is thus speculated that alternative adaptive emotion regulation strategies are suitable to implement in the context of an exclusive situation. More specifically, given the strong correlation between negative emotions and aggression [13], as well as the special role that anger appears to play in promoting aggressive behavior [14,15], it seems that the down-regulation of negative emotions, especially anger, is necessary to decrease the likelihood of aggressive behavior. 

Acceptance, as an adaptive emotion regulation strategy, is characterized by stressing that people maintain an open and receptive attitude towards their emotions and feelings without attempting to modify them [16]. This characteristic of the acceptance strategy is helpful for generating positive emotions, which in turn reduces an individual’s loneliness induced in social situations and then improves social relations [17,18,19]. In this sense, acceptance could be used as a useful strategy to alleviate negative consequences (emotions and/or aggressive behaviors) generated by social exclusion. Further evidence supporting this assumption indicates that people who habitually use acceptance strategy to cope with emotions in daily life experience fewer negative emotions (such as depression, anxiety and anger) [20,21,22,23,24,25,26]. In contrast, people with low trait acceptance exhibited more aggressive behavior toward violence [27,28,29] or dysfunctional attitudes toward environmental stress [30]. Nevertheless, few studies to date have explored whether the use of an acceptance strategy can lessen aggressive behavior by reducing negative emotions induced by social exclusion. 

**Figure 1:**
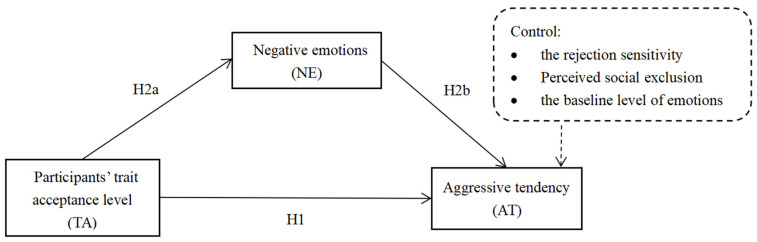
Hypothesized conceptual model.

Therefore, the current study sought to investigate whether emotion regulation in the context of social exclusion modulates negative emotions and aggressive behavior. To this end, the recall paradigm was utilized to induce social exclusion, trait acceptance was used as the emotion regulation strategy, and a questionnaire was used to gauge emotional states. In addition, it was discovered that the level of rejection sensitivity was significantly and positively correlated with aggressive behavior after rejection [31]. We also included rejection sensitivity as a control variable. Based on previous studies, we hypothesized that the trait acceptance level can significantly predict both aggression and negative emotions and that the negative emotions may act as a mediating factor between the trait acceptance level and aggressive tendency. The hypothesized conceptual model proposed in this study is shown in Figure 1.

**Hypothesis** **1** **(H1).** *TA is negatively related to AT.*

**Hypothesis** **2** **(H2).** *NE mediates the relationship between TA and AT.*

**Hypothesis** **2a** **(H2a).** *TA is negatively related to NE.*

**Hypothesis** **2b** **(H2b).** *NE is positively related to AT.*

## 2. Methods

### 2.1. Participants 

It was thought that undergraduate students were at high risk of suffering from social exclusion [32]; therefore, we used this group as a sample to explore the negative consequences of social exclusion and its regulation. In this study, a convenience sampling method was used to collect data from undergraduate students at Neijiang Normal University and Sichuan Normal University, and the questionnaires were collected using the Questionnaire Star online platform. A total of 468 participants completed questionnaires, and 71 of them had to be excluded because they did not fill out all scales as completely or carefully as required. As a result, 397 participants were included in the further data analysis [effective recovery rate: 85%; age range: 17–25 years old, M_age_ = 20.11 years, SD = 1.40; 309 female (77.83%)]. All the participants reported that they had no history of affective disorders or drug use. The participants who completed the questionnaire carefully (as assessed by the researchers) were compensated with CNY 5. The study was approved by the research team’s university ethics committee. 

### 2.2. Measurements

Experimental Task. Social exclusion was induced through the recall paradigm [33]. More specifically, participants were asked to carefully recall an experience of being rejected, isolated or ignored by a certain individual or group in recent months. Then, they were asked to write down not only the detailed process (e.g., when and how they were rejected or excluded) of this experience but also their own feelings and thoughts when these events happened to them. If the content involved private information, substitution with letters was allowed. After the recall task was completed, the participants were asked to rate the degree to which they felt rejected or excluded during this social exclusion experience, by rating on a 7-point scale ranging from 1—“nothing at all” to 7—“very strong”. The higher score indicates a greater extent of social exclusion experience.

Trait Acceptance. The Acceptance and Action Questionnaire-II (AAQ-II) [26] was used to measure trait acceptance. The scale consists of 7 items that measure experiential avoidance, which is opposed to trait acceptance [34]. Each item is rated on a 7-point scale ranging from 1—completely non-conforming—to 7—completely conforming. A higher score on this scale indicates a higher degree of experiential avoidance; namely, a lower degree of trait acceptance. The Cronbach’s alpha of the scale was 0.90 in this study.

Rejection Sensitivity. A Rejection Sensitivity Questionnaire (RSQ) was used to measure the rejection sensitivity, which is referred to as a tendency to expect and react strongly to rejection [35]. The RSQ is a 6-point scale including 16 items corresponding to 16 daily situations. It consists of two dimensions: rejection anxiety and accepted expectations. For the rejection anxiety subscale, 1 means “not worried at all” and 6 means “very worried”, while for the accepted expectations subscale, 1 means “completely impossible” and 6 means “very likely”. A higher score indicates a higher level of rejection sensitivity. The Cronbach’s alpha of RSQ was 0.92 in the current study.

Aggressive Tendency. The Aggression Questionnaire (AQ) [36] was used to assess the participants’ aggressive tendencies. This is a 29-item multidimensional scale. The four dimensions measured in this scale correspond to physical attack, verbal attack, anger, and hostility, respectively. Each item is rated on a 5-point scale ranging from 1—very non-conforming—to 7—very conforming. The higher score indicates a higher degree of aggression. The Cronbach’s α coefficient of the questionnaire in this study was 0.94. 

State emotion. The emotional scale used in this study combined the Positive and Negative Affect Scale (PANAS) [37] and the emotional items related to social rejection used in a study by Buckley et al. [38]. Specifically, this emotional scale consists of 30 emotional words, including 11 positive (Cronbach’s α = 0.95) words corresponding to active, enthusiastic, happy, elated, excited, proud, delighted, energetic, grateful, pleasant and cheerful dimensions, and 19 negative (Cronbach’s α = 0.97) words corresponding to anger (5 items: Cronbach’s α = 0.93), sadness (4 items: Cronbach’s α = 0.93), hurt feelings (4 items: Cronbach’s α = 0.90) and other negative emotions (6 items: Cronbach’s α = 0.93). Participants were asked to rate their emotion at the moment on a 7-point scale ranging from 1—“not at all”—to 7—“very strong”. See Appendix A for details.

Individual motivation to seek revenge. In this study, the 6-item Revenge subscale of the Transgression Related Interpersonal Motivations scale-18 items (TRIM-18) [39] was used to measure aggressive tendency. These 6 items are rated on a 5-point Likert-type scale (1 = strongly disagree to 5 = strongly agree). A higher total score indicates a higher level of aggressive tendency. In the present study, the Cronbach’s α coefficient of the scale was 0.90.

### 2.3. Procedure

After signing an informed consent form, the participants were asked to complete three personal trait scales, including the AAQ-II, RSQ and AQ. Then, they were asked to report their subjective feelings according to the emotional scale, which served as the baseline emotion state. After that, they started to seriously recall and write down their experiences of social exclusion and then reported their subjective feelings after the recall task.

### 2.4. Data Analysis

The data were analyzed using SPSS 25.0 statistical software. Based on the hypothesis of this study, we first conducted a descriptive analysis. After the scoring procedure, we analyzed the relationship between trait acceptance, emotions after social exclusion, and aggressive tendency by executing the Pearson correlation. Then, we explored the relationship between emotions and aggressive tendencies and trait acceptance using linear regression analysis. Lastly, PROCESS 3.3 in the SPSS software was utilized to compute the mediation models between trait acceptance, aggressive tendency and emotions after social exclusion, and the mediating effect was examined by performing a 5000 Bootstrap.

### 2.5. Assessment of Common Method Variance

To test the common method deviation or systematic measurement error caused by the self-described questionnaire collection of all the data, we carried out the Harman single-factor test [40], and an exploratory factor analysis was performed on all items. The results showed that the eigenvalues of 19 factors were larger than 1. The first factor explained 20.98% of the total variation, which was less than the 40% threshold criterion proposed by Podsakoff et al. [41]. This result suggests that common method variance is less likely to confound the primary results. 

## 3. Results

### 3.1. Correlation Analysis

The correlation results (see Table 1) showed that the AAQ score was significantly positively correlated with overall negative emotions (r = 0.41), anger (r = 0.32), sadness (r = 0.43), hurt feelings (r = 0.42) and other negative emotions (r = 0.33), *p* ≤ 0.001, whereas it was negatively correlated with positive emotions (r = −0.23, *p* < 0.001). Additionally, the AAQ score was significantly positively correlated with aggressive tendencies (i.e., revenge) (r = 0.33, *p* < 0.001).

### 3.2. Regression Analysis

When the emotion scores were used as dependent variables in the model (see Table A1 in Appendix A), it was found that the inclusion of the AAQ scores significantly increased the R^2^ of the regression model, where the dependent variables were overall negative emotions (ΔR^2^ = 0.03, *p* < 0.01), anger (ΔR^2^ = 0.02, *p* < 0.001), sadness (ΔR^2^ = 0.02, *p* < 0.001), hurt feelings (ΔR^2^ = 0.04, *p* < 0.001) and other negative emotions (ΔR^2^ = 0.02, *p* < 0.001). 

When the aggressive tendency scores were used as dependent variables in the model (see Table 2), it was found that the inclusion of the AAQ scores significantly increased the R^2^ of the model (ΔR^2^ = 0.02, *p* < 0.001). Moreover, after experiencing social exclusion, an individual’s trait acceptance level significantly negatively predicted their tendency to the ostracized (β = 0.17, *p* < 0.001).

### 3.3. Parallel Mediation Analysis

To further investigate whether trait acceptance influenced aggressive tendency through negative emotions induced by social exclusion, a parallel mediation analysis was conducted, with the baseline level of negative emotions and perceived social exclusion serving as covariables.

**Figure 2:**
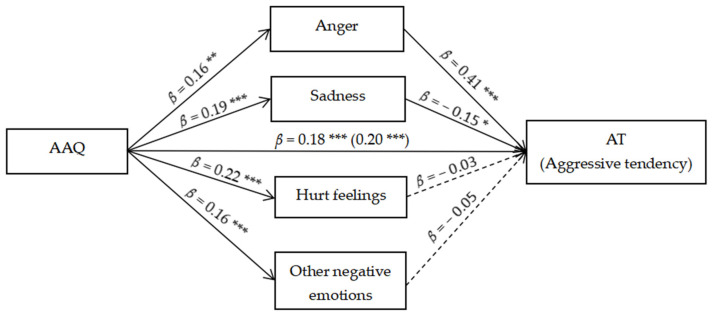
Parallel mediation model. * *p* < 0.05, ** *p* < 0.01, *** *p* < 0.001.

The results (see Table 3 and Figure 2) showed that the total effect (total effect = 0.205, SE = 0.039, 95% CI: [0.128, 0.281]) and direct effect (direct effect = 0.184, SE = 0.036, 95% CI: [0.113, 0.255]) of trait acceptance on aggressive tendency were both significant. Specifically, the relationship between trait acceptance and aggressive tendency was mediated by anger (Indirect 1 = 0.064, SE = 0.027, 95% CI: [0.012, 0.118]) and sadness (Indirect 2 = −0.029, SE = 0.015, 95% CI: [−0.063, −0.005]) after social exclusion. For path 1 (AAQ→Anger→AT), the ratio of these two effects to the overall effect was 31.22%, and for path 2 (AAQ→Sadness→AT), it was 14.15%. A further comparison regarding the size of the mediation effect (Indirect 1–Indirect 2 = 0.093, SE = 0.034, 95% CI: [0.029, 0.164]) showed that anger was the stronger mediator. However, hurt feelings (Indirect 3 = −0.006, SE = 0.015, 95% CI: [−0.039, 0.023]) and other negative emotions (Indirect 4 = −0.009, SE = 0.010, 95% CI: [−0.031, 0.011]) did not mediate the connection.

## 4. Discussion

In order to better understand the relationship between trait acceptance (as a method of emotional regulation) and aggressive tendencies in the setting of social exclusion, the current study looked into the role of emotions in mediating the relationship between these factors. The recall paradigm, which required participants to carefully recall and write a detailed experience of being rejected or isolated in recent months, was used to induce social exclusion, and the specific questionnaires were used to measure trait acceptance and aggressive tendency. As predicted, the results showed that trait acceptance negatively predicted not only negative emotions, but also aggressive tendencies. Moreover, the relationship between trait acceptance and aggressive tendency was mediated by anger and sadness.

As hypothesized, our results indicated that trait acceptance negatively predicted aggressive tendencies. It was thought that the acceptance strategy applied through the use of Acceptance and Commitment Therapy (ACT) can improve psychological flexibility [16,42,43], leading to the creation of broader and more flexible behavior options that could aid people in changing or eradicating negative or unwanted events [16,44,45], moving toward their predetermined life goals [44,45]. Considering that aggressive behaviors were found to be one of the “quick fix” solutions to ease negative emotions and unhappy memories caused by these events [27], we thus surmise that psychological flexibility is a possible factor that accounts for the predictive role of trait acceptance in aggressive tendencies. Alternatively, the inability to reduce negative emotions limits psychological flexibility somehow [16]. It thus makes sense to conceive that people with higher trait acceptance are characterized by higher psychological flexibility and are more likely to respond to social exclusion by engaging in adaptive rather than non-adaptive behavior (i.e., aggression). 

In addition, trait acceptance was a significant predictor of negative emotions and had a negative correlation with overall negative emotions. This was in line with existing studies showing a negative link between acceptance and negative emotions [20,21,46,47] and that the acceptance strategy was effective at reducing negative emotions [27,48,49] as an adaptive substitute for avoidance [50]. Further, it can be explained by the finding that higher trait acceptance was associated with improved emotion regulation abilities [23,48,49,51]. By this logic, it is believed that people who habitually use acceptance strategy in daily life are better able to cope with negative events, such as the social exclusion induced in this study, thus leading to fewer unpleasant feelings after rejection. In the present study, those with lower AAQ scores were more likely to utilize acceptance strategy to regulate negative emotions under social exclusion and, as a result, they felt fewer negative emotions.

The results also revealed that the association between trait acceptance and the aggressive tendency was mediated by anger. Anger, which is an approach-related affect [52], may lower psychological flexibility [53] by restricting behavioral options, according to other studies. According to this reasoning, anger may be the driving force for people’s aggressive behavior, and mitigating anger is thus essential in lowering aggression. This is in line with our finding that reducing anger through the use of acceptance strategy may be useful to lessen aggression. Specifically, those with lower AAQ scores that indicated a higher level of trait acceptance preferred to accept or embrace, rather than avoid or change, their feelings of anger during social exclusion. As a result, they exhibited a lower tendency for aggression. Despite this, determining whether acceptance strategy reduce aggressive behavior triggered by social exclusion through regulating anger still requires more experiment-based research.

Sadness also played a mediating role between trait acceptance and aggressive behavior. This suggested that people with higher trait acceptance levels show less sadness following rejection, which in turn increased their propensity for aggression. Compared to anger stemming from approach-related emotion, sadness arising from avoidance-related emotion activates an individual’s withdrawal system [54]. Thus, when people experienced sadness in the context of social exclusion, they tended to exhibit withdrawal behaviors (e.g., avoiding or leaving) rather than aggressive behaviors that are driven by anger. 

Further comparison results indicated that though both sadness and anger were found to mediate the association between trait acceptance and aggression, the effect size of the mediator was significantly higher for anger than for sadness. This suggested that compared to reducing sadness, the down-regulation of anger through the use of acceptance strategy was the main path to reduce aggression in the context of social exclusion. Indirect evidence for this conclusion came from a study showing that adolescents with high aggression levels or violent tendencies expressed greater anger but less sadness [44]. Therefore, using acceptance to regulate sadness would actually increase aggression as opposed to its success in reducing aggression by down-regulating anger. 

Several limitations warrant notice. First, although our focus was to investigate the predictive role of trait acceptance for negative emotions and aggressive behaviors, this study, however, merely examined acceptance strategy, without taking into account other adaptive emotion regulation strategies that potentially influence negative emotions and aggressive behaviors during social exclusion. Second, the majority of participants in this study were female. However, given that males (compared to females) express more sadness in reaction to rejection [50], a balanced gender ratio would provide more solid evidence for the mediating role of sadness in the association between trait acceptance and aggressive tendency. Lastly, the results of the current study were correlational in nature, other studies using longitudinal or experimental methods would be helpful to better understand and disclose the causal relationship between the above-mentioned variables. 

## 5. Conclusions

In summary, the current study suggests that trait acceptance is effective at alleviating negative emotions and lowering aggressive tendencies in the setting of social exclusion. Specifically, higher trait acceptance predicted lower aggression by down-regulating the anger of rejected individuals. These results contribute to our understanding of how individual differences in the habitual use of acceptance strategy influence the emotional state and, subsequently, aggression induced by social exclusion. The results also provide a preliminary basis for further use of acceptance strategy or acceptance-based interventions to alleviate negative emotional experiences and maladaptive behaviors in interpersonal frustration situations.

## Figures and Tables

**Table 1:** Correlation results between trait acceptance, aggressive tendency and emotions.

Variable	*M*	*SD*	1	2	3	4	5	6	7	8
1 AAQ	4.13	1.27	1							
2 Overall negative emotions	2.76	1.30	0.41 ***	1						
3 Anger	2.71	1.46	0.32 ***	0.87 ***	1					
4 Sadness	3.18	1.60	0.43 ***	0.90 ***	0.68 ***	1				
5 Hurt feelings	3.03	1.57	0.42 ***	0.93 ***	0.73 ***	0.91 ***	1			
6 Other negative emotions	2.36	1.29	0.33 ***	0.88 ***	0.68 ***	0.68 ***	0.73 ***	1		
7 Positive emotions	2.79	1.40	−0.23 ***	0.08	0.10 **	−0.08	−0.06	0.28 **	1	
8 Aggressive tendency	2.78	0.98	0.33 ***	0.36 ***	0.48 ***	0.25 ***	0.30 ***	0.23 ***	0.02	1

AAQ: scores on the Acceptance and Action Questionnaire-II, the same below; ** *p* < 0.01, *** *p* < 0.001. M: mean value; SD: standard deviation.

**Table 2:** The results of the hierarchical multiple regression analysis of aggressive tendency with trait acceptance.

Dependent Variables	Predictor	Step 1			Step 2		
		*b*	*SE*	*β*	*b*	*SE*	*β*
**Aggressive tendency**	Perceived social exclusion	0.22	0.03	0.31 ***	0.19	0.03	0.27 ***
	RSQ	−0.03	0.01	−0.11 *	−0.04	0.01	−0.15 **
	AQ	0.53	0.07	0.36 ***	0.44	0.07	0.30 ***
	AAQ				0.13	0.04	0.17 **
	ΔR^2^	0.26 ***			0.02 ***		

AAQ: scores on the Acceptance and Action Questionnaire-II; RSQ: scores on the Rejection Sensitivity Questionnaire; AQ: scores on the Aggression Questionnaire; b: the non-normalized coefficient, and *β*: the normalized coefficient; * *p* < 0.05, ** *p* < 0.01, *** *p* < 0.001.

**Table 3:** The results of parallel mediation analysis of negative emotions following social exclusion.

Paths	Indirect Effects	Boot SE	Boot LLCI	Boot ULCI	Relative Mediation Effect
Total effect: AAQ→AT	0.205	0.039	0.128	0.281	-
Direct effect: AAQ→AT	0.184	0.036	0.113	0.255	-
Total indirect effects					
Ind1: AAQ→Anger→AT	0.064	0.027	0.012	0.118	31.22%
Ind2: AAQ→Sadness→AT	−0.029	0.015	−0.063	−0.005	14.15%
Ind3: AAQ→Hurt feelings→AT	−0.006	0.015	−0.039	0.023	-
Ind4: AAQ→Other negative emotions→AT	−0.009	0.010	−0.031	0.011	-
Compare1:Ind1-Ind2	0.093	0.034	0.029	0.164	-

AAQ: scores on the Acceptance and Action Questionnaire-II; AT, Aggressive tendency; the same below.

## Data Availability

The data presented in this study are available on request from the corresponding author. The data are not publicly available due to privacy restrictions.

## Appendix A. Hierarchical Multiple Regression Results

**Table A1:** The results of hierarchical multiple regression analysis of emotions after experiencing social exclusion to the trait acceptance.

Dependent Variables	Predictor	Step 1			Step 2		
		*b*	*SE*	*β*	*b*	*SE*	*β*
**Overall negative emotions**	Baseline	0.66	0.04	0.62 ***	0.61	0.04	0.58 ***
	Perceived social exclusion	0.25	0.03	0.27 ***	0.21	0.03	0.22 ***
	RSQ	−0.01	0.01	−0.03	−0.03	0.01	−0.08
	AAQ				0.19	0.04	0.19 ***
	ΔR*	0.51 ***			0.03 **		
Anger	Baseline	0.59	0.05	0.50 ***	0.56	0.05	0.47 ***
	Perceived social exclusion	0.32	0.04	0.30 ***	0.28	0.04	0.26 ***
	RSQ	−0.03	0.02	−0.07	−0.05	0.02	−0.12 **
	AAQ				0.19	0.05	0.16 ***
	ΔR*	0.37 ***			0.02 ***		
Sadness	Baseline	0.63	0.04	0.58 ***	0.58	0.04	0.53 ***
	Perceived social exclusion	0.34	0.04	0.30 ***	0.30	0.04	0.26 ***
	RSQ	−0.01	0.02	−0.03	−0.03	0.02	−0.07
	AAQ				0.22	0.05	0.17 ***
	ΔR*	0.49 ***			0.02 ***		
Hurt feelings	Baseline	0.61	0.05	0.52 ***	0.55	0.05	0.47 ***
	Perceived social exclusion	0.35	0.04	0.31 ***	0.29	0.04	0.26 ***
	RSQ	−0.02	0.02	−0.04	−0.04	0.02	−0.10 **
	AAQ				0.28	0.05	0.22 ***
	ΔR*	0.43 ***			0.04 ***		
Other negative emotions	Baseline	0.72	0.04	0.69 ***	0.70	0.04	0.67 ***
	Perceived social exclusion	0.09	0.03	0.09 *	0.05	0.03	0.06
	RSQ	0.01	0.01	0.04	0.001	0.13	0.001
	AAQ				0.14	0.04	0.14 ***
	ΔR*	0.51 ***			0.02 ***		
Overall positive emotions	Baseline	0.70	0.04	0.64 ***	0.69	0.04	0.63 ***
	Perceived social exclusion	−0.20	0.04	−0.20 ***	−0.17	0.04	−0.17 ***
	RSQ	0.02	0.01	0.05	0.03	0.02	0.07
	AAQ				−0.09	0.05	−0.08
	ΔR*	0.43 ***			0.01		

AAQ: scores on the Acceptance and Action Questionnaire-II; RSQ: scores on the Rejection Sensitivity Questionnaire; b: the non-normalized coefficient, and *β*: normalized coefficient; * *p* < 0.05, ***p* < 0.01, *** *p* < 0.001.

## Appendix B. Component Words the Emotion Scale

Please choose the number to which each of the adjectives below describes how you feel at the moment. 1 = “very little or none”, 4 = “moderate” and 7 = “very strong”, with higher numbers indicating higher levels.

Positive Emotions (11): active, enthusiastic, happy, elated, excited, proud, delighted, energetic, grateful, pleasant, cheerful;

Anger (5): irascible, annoyed, irritated, angry, furious;

Sad (4): sad, depressed, sorrow, depressed;

Hurt Feelings (4): heartache, pain, sting, heart hurt;

Other Negative Emotions (6): ashamed, afraid, nervous, frightened, guilty, trepidation.
